# Comparison of the new Japanese legislation for expedited approval of regenerative medicine products with the existing systems in the USA and European Union

**DOI:** 10.1002/term.2428

**Published:** 2017-06-27

**Authors:** Yoji Jokura, Kazuo Yano, Masayuki Yamato

**Affiliations:** ^1^ Joint Graduate School of Tokyo Women's Medical University and Waseda University Tokyo Japan; ^2^ Institute of Advanced Biomedical Engineering and Science Tokyo Women's Medical University Tokyo Japan; ^3^ Research Institute for Science and Engineering Waseda University Tokyo Japan

**Keywords:** regulatory approval, expedited‐approval pathway, regenerative medicine product, pre‐marketing, post‐marketing, unmet medical need

## Abstract

Legislation for expedited‐approval pathways and programmes for drugs, biologics or medical devices has been enacted for rapid commercialization of innovative products in the United States of America (USA) and the European Union (EU). However, less innovative products are increasingly benefitting from these expedited‐approval pathways, and obligations to collect and report post‐marketing data on approved products are being bypassed frequently. The Japanese government recently enacted legislation for a new conditional and time‐limited approval pathway dedicated to regenerative medicine products. The current study examines this new legislation and compares it with existing US and EU regulatory frameworks, with a particular focus on how it addresses the limitations of existing systems. Regulations, guidance documents and approval information were gathered from the websites of the respective authorities in the USA, the EU and Japan, and the systems were categorized through qualitative analysis. The pathways and programmes from each region were categorized into four groups, based on the requirement of pre‐ or post‐marketing clinical data. Expedited‐approval pathways in the USA and the EU provide similar qualification criteria, such as severity of target disease; however, such criteria are not specified for the new pathway in Japan. Only the Japanese pathway stipulates a time limitation on exceptional approval, requiring post‐marketing study for conditional and time‐limited products. Continuous improvement is necessary to solve previously addressed issues within the expedited‐approval pathways and programmes and to ensure that innovative medical products are rigourously screened, but also readily available to patients in need. The time limitation of conditional approval could be a potential solution to some of these problems. Copyright © 2017 The Authors. *Tissue Engineering Regenerative Medicine* published by John Wiley & Sons, Ltd.

## 1 Introduction

Unmet medical needs demand the further development of truly innovative new medical products and treatment options, including regenerative medicine products. Expedited‐approval pathways and programmes, priority review or programmes alternative to the standard review processes for medical products have been developed and legislation has been enacted for the rapid commercialization of innovative medical products and for improved access for patients in need (US Food and Drug Administration, 2014c), (European Medicines Agency, 2016b). Some of these alternative pathways and programmes reduce the evidentiary requirement in the pre‐marketing development of medical products, which typically require demonstration of safety, efficacy or provision of risk‐benefit confirmatory data before product commercialization in the standard review process, and rather, make these evidentiary requirements post‐marketing obligations, such as the submission of post‐marketing clinical study data to the appropriate authorities.

Since the late 1980s, multiple alternative pathways or programmes have been introduced in the United States of America (USA) and the European Union (EU), and in the past two decades, many medical products (drugs, biologics and medical devices) have thus been approved (United States Government Accountability Office, 2015c; Boucaud‐Maitre and Altman, 2016). In the last decade, regulatory issues associated with these expedited‐approval pathways or programmes have arisen and need to be addressed. In the USA, an increasingly large number of minimally innovative or clinically transformative therapeutics are benefitting from the expedited development and review programmes created by the US Food and Drug Administration (FDA) (Kesselheim *et al.*, 2015). A large number of obligations to collect and report post‐marketing data on approved drugs have been bypassed by sponsors (Moore and Furberg, 2014). In the EU also, there have been delays or discrepancies in fulfilling post‐marketing obligations in more than one third of granted conditional approvals (Banzi *et al.*, 2015). Objections by the US congress or patient advocacy groups have reversed withdrawal decisions previously made by the legislative authorities (Dhruva and Redberg, 2010).

Recently, the Japanese government reformed its pharmaceutical affairs legislation, which regulates all pharmaceutical products and medical devices, and created a new regulation called the Pharmaceuticals, Medical Devices and Other Therapeutic Products Act (PMD Act) in November 2014 (Hara*, et al.*, 2014). Under this new Act, regenerative medicine products are classified independently from conventional pharmaceuticals and medical devices and are defined as processed human cells that are intended to be used 1) for either (a) the reconstruction, repair or formation of structures or functions of the human body or (b) the treatment or prevention of human diseases, or 2) for gene therapy.

This new Act also introduces conditional and time‐limited approval for regenerative medicine products, and one product has already been granted conditional and time‐limited authorization based on the probable benefit that was demonstrated by pilot clinical trial data. Critics claim that this new system would enable the approval of medical products with unconfirmed efficacy (Nature Editorials, 2015; Sipp, 2015). Because the first approved medical product is covered by Japanese national health insurance, critics also claim that insurance organizations and patients will be taking on some of the risk and financial burden of phase III studies that should instead be shouldered by the sponsor to confirm product safety and efficacy (McCabe and Sipp, 2016). Aside from commentaries or letters, to our knowledge, there are no published studies that examine or analytically compare the legislative framework underlying each of these various regulations. In this study, we first compare the expedited‐approval systems in the USA and EU with the new approval pathway for regenerative medicine products in Japan, and then propose points for improvement of future systems.

## 2 Materials and methods

### 2.1 Categorization of the US and EU expedited‐approval pathways or programmes and comparison with the new conditional and time‐limited approval of regenerative medicine products in Japan

Given the objective of the study, to compare the new expedited‐approval pathway in Japan with those existing in the USA and the EU, we selected 10 pathways and programmes in the USA and the EU that fulfill either of the following two conditions:

1. ‘Standard’ approval is provided through an expedited review process;
2. Exemption from demonstration of clinical effectiveness and conditional approval are granted.

All regulation documents pertaining to expedited approval of medical products were obtained from websites of the respective authorities: the FDA (US Food and Drug Admministration, 2016e), the European Medicines Agency (EMA) (European Medicines Agency, 2016a) and the Pharmaceuticals and Medical Devices Agency (PMDA) (Pharmaceuticals and Medical Devices Agency, 2016a). Requirements related to collecting post‐marketing clinical data were also obtained through guidance documents or legislation. Information on the regulatory pathways and programmes in the USA and EU was categorized according to the following criteria: pre‐ and/or post‐marketing data requirements, evaluation of post‐marketing data, and existence of regulatory authority development support. Then, qualifying criteria (Table 3) of the USA and the EU pathways and programmes were identified and displayed to facilitate comparison with the new pathway in Japan.

### 2.2 Analysis of post‐marketing clinical data requirements for approved regenerative medicine products in Japan

In addition to regulation documents, the review report of two products, HeartSheet® (Ministry of Health, Labor and Welfare, 2015a) and Temcell HS Injection® (Ministry of Health, Labor and Welfare, 2015b), which have been approved under the new PMD Act as of the end of September 2016 (Pharmaceuticals and Medical Devices Agency, 2016b) were obtained from the PMDA website. The clinical data submitted for obtaining marketing authorization and post‐marketing obligations were analysed.

## 3 Results

### 3.1 Categorization of expedited‐approval pathways and programmes in the USA and EU

Table 1 summarizes the identified the pathways and programmes in the US and the EU for expedited approval or priority review of the medical products. Based on the criteria described in the Methods section, the pathways and programmes (Table 1) were categorized into the following four groups (Table 2):

1. Expedited approval without post‐marketing obligations;
2. Conditional approval with evaluation of required post‐marketing data after approval;
3. Approval with limited patient cohort or indication with no confirmed efficacy data required for marketing approval and no post‐marketing obligations;
4. Development support by the authority for expedited product approval.

**Table 1:** Expedited‐approval pathways and programmes in the USA and EU

**Regulatory pathway (Authority, year of issue)**	**When to submit request and timeline for authority response**	**Qualifying criteria** **(Scope and requirements)**	**Features and mechanism of expedition**	**Post‐marketing data requirement**
**USA**				
Priority review (FDA, 1992)	Submit with original BLA, NDA, or efficacy supplement Response within 60 calendar days of receipt of submission	1) A drug for a serious condition, AND with a significant improvement in safety or effectiveness, OR 2) Supplement for a labeling change pursuant to a report on a pediatric study, OR 3) A drug designated as a qualified infection disease product (QIDP) OR 4) Submitted with a priority review voucher	Shorter review time of marketing application (6 months, compared with 10‐month standard review)	Not requested
Accelerated approval (FDA, 1992)	Submit during drug development Response timing not specified	1) A drug for a serious condition, AND 2) With a meaningful advantage over available therapies, AND 3) Demonstrates an effect • on a surrogate endpoint that is reasonably likely to predict clinical benefit, OR • on an intermediate clinical endpoint that is reasonably likely to predict clinical benefit	Approval based on effect on a surrogate endpoint or an intermediate clinical endpoint that is reasonably likely to predict a drug's clinical benefit	Post‐marketing confirmatory trials to verify and describe the anticipated effect on IMM or other clinical benefit
Fast track (FDA, 1998)	Submit with IND or after; ideally, no later than the pre‐BLA or pre‐NDA meeting Response within 60 calendar days of receipt of request	1) A drug for a serious condition AND nonclinical or clinical data with the potential to address unmet medical need OR 2) A drug designated as a QIDP	Actions to expedite development and review; Rolling review	Not requested
Breakthrough therapy (FDA, 2012)	Submit with IND or after; ideally no later than the end‐of‐phase‐2 meeting Response within 60 calendar days of receipt of request	A drug for a serious condition AND preliminary clinical evidence with substantial improvement on a clinically significant endpoint(s) over available therapies	Intensive guidance on efficient drug development; Organizational commitment; Rolling review	Not requested
Expedited access pathway (FDA, 2015)	Submit prior to commencement of an IDE pivotal study Meeting will be held within 75‐90 days of request with pre‐submission by the sponsor	A device intended to treat or diagnose a life‐threatening or irreversibly debilitating disease or condition AND addresses an unmet need	Reduces premarket data	Post‐approval data collection may be required for conditional approval (data collection should begin within 6 months of, and be submitted within 3 years of, approval data)
**EU**				
Accelerated assessment (EMA, 2004)	Submit request at least 2‐3 months before submission of marketing authorization application (MAA) Conclusions on request for accelerated assessment communicated to applicant prior to start of product assessment.	Medicinal products of major interest to public health AND therapeutic innovation perspective (unmet medical need)	Reduced MAA assessment time to 150‐day maximum compared to standard 210 days	Not requested
Marketing authorization under exceptional circumstances (EMA, 2005)	Submit before MAA No response timeframe specified	1) Indications encountered so rarely that the applicant cannot reasonably be expected to provide comprehensive evidence, OR 2) In the present state of scientific knowledge, comprehensive information cannot be provided, OR 3) It would be contrary to generally accepted principles of medical ethics to collect such information	Authorization without comprehensive data on efficacy and safety	The applicant shall complete an identified programme of studies within the time period specified by the competent authority, the results of which shall form the basis of a reassessment of the benefit/risk profile (Does not normally lead to standard authorization)
Conditional marketing authorization (EMA, 2006)	Request at submission of MAA. The CHMP also proposed a conditional marketing authorization during assessment of MAA Assessed as part of the scientific review during assessment of MAA	Medical products for: • Seriously debilitating or life‐threatening diseases • Emergency situations • Orphan medicinal products, AND Fulfilling all of the following criteria: • Positive risk‐benefit balance • Applicant likely to be able to provide comprehensive data after authorization • Fulfillment of unmet medical need • Benefits of immediate availability outweigh the risks that additional data are still required	Earlier authorization of medicines for patients with unmet medical needs, based on less complete clinical data	Comprehensive data generated post‐authorization within agreed timeframe (Valid for one year; thereafter, conditional marketing authorization may be renewed annually)
Adaptive pathway approach (EMA, 2015)	Contact EMA to discuss the draft content and determine suitability of their request to be considered for the pilot programme case‐by‐case No response timeframe specified	Treatments in area of high medical need where it is difficult to collect data via traditional routes and where large clinical trials would unnecessarily expose patients who are unlikely to benefit from the medicine	Scientific advice by the authority; Compassionate use; The conditional approval mechanism	Patient registries and other pharmacovigilance tools that allow collection of real‐life data and development of a risk‐management plan
Accelerated assessment of priority medicines, PRIME (EMA, 2016)	Submit during the drug development, based on preliminary clinical evidence (proof of concept); Exceptionally earlier access to SMEs and academics (proof of principle); at least first in man	Fulfills unmet clinical need	Identify potential for accelerated assessment earlier in development: − Early rapporteur appointment − Reinforced scientific and regulatory support from the SAWP/CHMP, other relevant scientific committees, and EMA − Dedicated contact person within EMA	N/A

BLA, Biologics License Application; CHMP, Committee for Human Medicinal Products; EMA, European Medicines Agency; EU, European Union; FDA; Food and Drug Administration; IMM, irreversible morbidity or mortality; IND, Investigational New Drug; IDE, Investigational Device Exemption; MAA: Marketing Authorization Application; NDA, New Drug Application; USA, United States of America.

**Table 2:** Categorization of expedited or special pathways in the USA and EU

**Category**	**Expedited pathway or programme (Authority)**	**Requirement of efficacy/benefit‐risk balance clinical data**		**Review/ evaluation of post‐marketing data**
		**Pre‐marketing data**	**Post‐marketing data**	
1. Expedited approval (with no conditions)	‐ Priority review (FDA) ‐ Fast track (FDA) ‐ Accelerated assessment (EMA)	Comprehensive data are required	Not required	Not required
2. Conditional approval	‐ Accelerated approval (FDA) ‐ Expedited approval pathway (FDA) ‐ Conditional marketing authorization (EMA)	Comprehensive data are not required Early, surrogate, or intermediate endpoints are accepted	Required	Required
3. Approval with limited patient cohort or indication	‐ MA under exceptional circumstances (EMA)	Not required	Not required	Not required
4. Development support	‐ Breakthrough therapy (FDA) ‐ Adaptive pathways (EMA) ‐ PRIME (EMA)	Comprehensive data are required	Not required (except for adaptive pathway)	Not required

EMA, European Medicines Agency; EU, European Union; FDA; Food and Drug Administration; USA, United States of America.

If the new Japanese expedited‐approval pathway were to be categorized into one of the above groups, it would fit into group 2.

### 3.2 Qualifying criteria for expedited‐approval pathways and programmes in the USA and EU, and comparison with those of the conditional and time‐limited approval of regenerative medicine products in Japan

Based on the guidance documents for the various US and EU pathways and programmes, the following qualifying criteria for candidate medical products were found to be common between two or more pathways or programmes. The candidate medical product:

- Treats a serious or life‐threatening target disease;
- Treats a limited target disease patient population (e.g. orphan disease);
- Treats a disease lacking medical treatment options (e.g. unmet medical needs);
- Is superior to existing medical treatment options.

These conditions are summarized in Table 3 (✓, pathways/programmes that include these criteria; ‐, those without these criteria). Briefly, most pathways had a requirement that the candidate medical product should address unmet medical needs. Almost two thirds of the examined expedited‐approval pathways or programmes required that the products were intended for serious or life‐threatening diseases. All of the analysed pathways include one or more of these four criteria.

**Table 3:** Summary of qualifying criteria for expedited pathways/programmes for medical products

**Regulatory pathway (Authority)**	**Serious/life‐threatening target disease**	**Limited/orphan patient population**	**No alternative treatment options**	**Superior to existing treatment**
Priority review (FDA)	✓^a^	–b	–	✓
Accelerated approval (FDA)	✓	–	–	✓
Fast track (FDA)	✓	–	✓	–
Breakthrough therapy (FDA)	✓	–	–	✓
Expedited access pathway (FDA)	✓	–	✓	–
Accelerated assessment (EMA)	–	–	✓	–
Marketing authorization under exceptional circumstances (EMA)	–	✓	–	–
Conditional marketing authorization (EMA)	✓	✓	✓	–
Adoptive pathway (EMA)	–	✓	✓	–
PRIME (EMA)	–	–	✓	–

a : Required.

b : Not required.

FDA, US Food and Drug Administration; EMA, European Medicines Agency

We analysed the applicable provisions of the conditional and time‐limited approval of the PMD Act. (Ministry of Welfare, Health and Labor, 2013) Part 1, Articles 23‐26 of the Act is the only portion containing similar qualifying criteria, as outlined below:

1. The regenerative medicine product is not homogeneous;
2. The clinical data on the products are likely to predict efficacy;
3. The product does not exhibit remarkably adverse results in efficacy, effectiveness or performance.

There is no definition of ‘homogeneous’ in the legislation; this designation for conditional and time‐limited approval is to be determined based on consultation with the Pharmaceutical Affairs and Food Sanitation Council (Part 1, Article 23‐26 of the Act).

### 3.3 Japanese standards for collecting post‐marketing clinical data under conditional and time‐limited approval

After obtaining conditional and time‐limited approval, the marketing authorization holder must submit a standard authorization application with data collected from all patients treated with the product over the allocated conditional approval period. The quality and compliance requirements fulfilled for post‐marketing study of conditional and time‐limited approved products is the Good Post‐marketing Study Practice (GPSP) (Ministry of Welfare, Health and Labor, 2014b) which is usually applied for post‐marketing surveillance (PMS) (Part 5, Article 23‐26 of the Act). PMS is generally required for ‘novel’ medical products (no similar or predicated product approved in Japan). In contrast, the clinical data submitted to the regulatory authority for pursuing the standard approval must comply with Good Clinical Practice (GCP) (Part 3, Article 23‐25 of the Act) (Ministry of Welfare, Health and Labor, 2014a).

### 3.4 Clinical data for conditional and time‐limited approval and post‐marketing clinical data requirements

As of the of the end of September 2016, two regenerative medicine products have been approved under the new Japanese Act (Pharmaceuticals and Medical Devices Agency, 2016c, 2016d). One of them, HeartSheet® (Terumo), was granted conditional and time‐limited approval on 18 September 2015. The product is made of autologous skeletal myoblasts, and is used to treat patients with serious heart failure (Ministry of Health, Labor and Welfare, 2015a). The applicant medical product demonstrated probable benefit in one multi‐centre, open‐label, single‐arm, feasibility study with seven patients (Ministry of Health, Labor and Welfare, 2015a). To obtain standard approval, a standard authorization application with data collected from all cases treated with the product over the allocated conditional approval period, which was five years, must be submitted. Although the maximum time of conditional approval is regulated as seven years (Part 1, Article 23‐26 of the Act), the reasons for the five‐year approval were not described in the review report. Patients enrolled into the study were 60 for the product arm and 120 for the control arm, not treated with the product, but identified with similar clinical conditions.

The second product, TemCell HS Injection® (JCR Pharmaceuticals Co. Ltd.), was approved on September 18, 2015 (Ministry of Health, Labor and Welfare, 2015b). Unlike HeartSheet, it was granted standard approval. It is an allogenic cell therapy product, and intended to treat an orphan disease, acute graft‐versus‐host disease (aGVHD) after an allogeneic bone marrow transplant. Unlike the case with HeartSheet, one condition for approval is collecting data on all cases treated with the product, which should be registered as PMS.

## 4 Discussion

Through comparative analysis, the following three major differences between the new Japanese system and the existing US and EU systems were identified, and we focus on these in our discussion.

1. The new Japanese expedited‐approval system does not specify the severity of the target disease, or patient population, or make comparisons with existing treatment conditions or requirements for conditional and time‐limited approval, as in the expedited‐approval pathways and programmes in the USA and EU.
2. The Japanese system contains a time‐limited (maximum seven years) and conditional approval, while the USA and EU have no time limitations on any marketing of specialized drug approvals.
3. The standard required for post‐marketing study in Japan is a unique practice, and is specialized for data collection in the post‐marketing setting.

### 4.1 Qualifying criteria for the expedited‐approval pathways and programmes

We have clarified that the Act in Japan does not specify the qualifying criteria for conditional and time‐limited approval, as is the case for the expedited‐approval pathways and programmes in the USA and EU. Furthermore, under the new Act, designation of the programme is determined during the review process by consulting an external review board, meaning that decisions are made at a late stage in the review process as compared to the US and EU systems (Ministry of Welfare, Health and Labor, 2013). However, since only two products have been approved through the new Japanese system, comparisons between these products can be made based on the collective characteristics of their procedures and characteristics. According to our analysis of the HeartSheet review report the target disease was severe chronic heart failure, a serious and life‐threatening condition, and there was no realistic alternative medical treatment. However, these conditions were not detailed in the legislation. Without clarifying whether the data from phase II studies (or pilot studies) are likely to be accepted under the expedited approval as specified in the Act, it is challenging for applicants or sponsors to formulate a development plan in advance without clear guidance. In addition to institutionalization of the qualifying criteria, considering the fact that recent applications have not always met qualifying criteria in the USA (Kesselheim*, et al.*, 2015), mechanisms for preventing the applications that fall outside specified programme conditions should be established.

### 4.2 Advantage of time‐limitation of marketing authorization

In the USA, sponsors do not sufficiently uphold and complete their obligation to collect post‐marketing data (Moore and Furberg, 2014). Concerning expedited‐approval programmes in the EU, it has been reported that there were delays or discrepancies in the fulfilment of the post‐market obligations in more than one third of the conditional approvals (Banzi *et al.*, 2015). Moreover, as in the example of Midodrine in the USA (Dhruva and Redberg, 2010), it has sometimes become difficult for authorities to withdraw products of questionable efficacy from the market because of appeals made by professional organizations, health care professionals and patients.

Compared with these concerning complications in the US and EU programmes, the time‐limitation of marketing approval for regenerative medicine products in Japan is unique, enabling conditional approval to be automatically withdrawn if the applicant or sponsor cannot obtain standard approval during the allocated period. This additional requirement is intended to improve sponsor compliance with post‐marketing obligations.

### 4.3 Standard or practice requirements for post‐marketing study of conditional and time‐limited approval

As described in the Results section, GPSP is the required standard for conducting PMS for conditional and time‐limited approval products. GPSP is a unique Japanese standard applied to PMS and required for unique medical products without precedent approvals. For example, monitoring and self‐auditing are imperative under GCP, but are not required under GPSP. However, under the new Japanese legislation the authority conducts a conformity audit after data submission for regulatory purposes. Therefore, GPSP applies a less rigid standard in terms of data reliability. Practically, it would become more challenging to conduct clinical trials (studies) to determine efficacy before approval because of the difficulty of enrolling a sufficient number of patients (Darrow *et al.*, 2014). Since PMS confirmatory clinical data are required to be submitted by the end of the limited permitted marketing period, it seems counter‐intuitive to apply criteria less rigourous than those for the standard approval conducted under GCP. As HeartSheet treats serious heart failure, it would be challenging for the sponsor to design studies to obtain confirmatory efficacy data from post‐marketing studies where finding control or placebo groups could be difficult. Given that a product's effectiveness should be proven by a post‐marketing study or by surveillance, additional mechanisms for using such non‐GCP study data to confirm safety and efficacy should be implemented.

Both the time limitation for expedited approval and the standards set for post‐marketing surveillance are unique systems in Japan that would be another solution for more effectively enforcing compliance with post‐marketing obligations. There are many variations in expedited‐approval pathways and programmes in the USA, the EU and Japan; however, efforts to improve the respective systems should focus on solving the problems unique to each existing framework.

## 5 Conclusions

The expedited‐approval pathways and programmes in the USA, the EU and Japan play a key regulatory role in accelerating the development of medical products, including regenerative medicine products for unmet medical needs. Improvements in the expedited‐approval pathways and programmes are necessary to solve the problems inherent in the existing systems. Our study shows that the characteristics of the new Japanese legislation may improve institutionalization of the other expedited‐approval pathways and programmes.

## Conflict of interest

Y. J. is an employee of Cook Japan Inc. K. Y. is an employee of Medtronic Japan Co., Ltd. M. Y. is a shareholder of CellSeed Inc. No funding was utilized for this study.
